# Analysis of Bladder Cancer Staging Prediction Using Deep Residual Neural Network, Radiomics, and RNA-Seq from High-Definition CT Images

**DOI:** 10.1155/2024/4285171

**Published:** 2024-04-30

**Authors:** Yao Zhou, Xingju Zheng, Zhucheng Sun, Bo Wang

**Affiliations:** ^1^Department of Radiology, Affiliated Hospital of Guizhou Medical University, Guiyang, China; ^2^Department of Radiology, Guizhou Provincial People's Hospital, Guiyang, China; ^3^Interventional Radiology, Guizhou Provincial People's Hospital, Guiyang, China

## Abstract

Bladder cancer has recently seen an alarming increase in global diagnoses, ascending as a predominant cause of cancer-related mortalities. Given this pressing scenario, there is a burgeoning need to identify effective biomarkers for both the diagnosis and therapeutic guidance of bladder cancer. This study focuses on evaluating the potential of high-definition computed tomography (CT) imagery coupled with RNA-sequencing analysis to accurately predict bladder tumor stages, utilizing deep residual networks. Data for this study, including CT images and RNA-Seq datasets for 82 high-grade bladder cancer patients, were sourced from the TCIA and TCGA databases. We employed Cox and lasso regression analyses to determine radiomics and gene signatures, leading to the identification of a three-factor radiomics signature and a four-gene signature in our bladder cancer cohort. ROC curve analyses underscored the strong predictive capacities of both these signatures. Furthermore, we formulated a nomogram integrating clinical features, radiomics, and gene signatures. This nomogram's AUC scores stood at 0.870, 0.873, and 0.971 for 1-year, 3-year, and 5-year predictions, respectively. Our model, leveraging radiomics and gene signatures, presents significant promise for enhancing diagnostic precision in bladder cancer prognosis, advocating for its clinical adoption.

## 1. Introduction

Cancer of the urothelium lies between the renal pelvis and the urethra, accounting for approximately 3 percent of all cancer-related deaths in the United States [1]. Two different risk factors are associated with this disease that most commonly occur in the bladder [2]. In the Western world, smoking and exposure to environmental and industrial carcinogens pose the most serious health risks [3]. There are two distinct but somewhat overlapping pathways in the development of bladder cancer, termed papillary and nonpapillary, corresponding to two distinctly clinical and pathogenetically distinct types [4]. There are approximately 80% of bladder neoplasms that are superficial papillary lesions caused by diffuse mucosal hyperplastic changes known as low-grade urothelial neoplasia [5]. It is still difficult to predict the outcome for patients with advanced or chemotherapy-resistant bladder cancer, despite progress in surgical techniques and drug therapy [6].

For the diagnosis of bladder cancer, computed tomography (CT) is the most common method [7]. When bladder cancer is diagnosed preoperatively, preoperative staging can be more accurate, and recurrence can be detected earlier after surgery with preoperative diagnosis [8]. The acquisition of traditional medical CT examination images, on the other hand, requires a great deal of time and space since more information is contained in them about the human tissues [9]. It is therefore not only more difficult but also more expensive to segment CT images using CT image segmentation technology [10]. In medical CT images, it is easy to cause mis-segmentation, especially when body tissues have abnormalities, such as severely damaged tissues [11].

Over the past few years, radiomics has gained more and more attention. Medical images are converted into high-dimensional, mineable data using high-throughput quantitative feature extraction, followed by data analysis to support decision-making [12]. As pattern recognition tools and dataset sizes have grown, radiomics has made progress, which may improve oncology prediction accuracy [13]. A number of previous studies have demonstrated the potential of objective and quantitative imaging descriptors as prognostic and predictive biomarkers [14].

An RNA-sequencing study measures the mRNA, small RNA, noncoding RNA, and other expression levels in a transcriptome by using the high-throughput sequencing technology. Since the early 2000s, the RNA-Seq technology has grown rapidly and has become one of the most essential tools for analyzing transcriptome-wide gene expression changes and alternative splicing of mRNAs. It has become possible to apply RNA-Seq technology to a broader range of applications with the development of next-generation sequencing technology. Multiple biomarkers from the RNA-sequencing study can guide the diagnosis and treatment of cancer patients. As a marker powerful enough to transform clinical management, a panel of biomarkers rather than their individual analyses provides the most promising approach. Therefore, in this work, we aim to construct the model based on the RNA-sequencing analysis and radiomics for the better prediction of the prognosis and the treatment of bladder cancer patients. In addition, we also evaluate the immune cell infiltration analysis based on the combination of RNA-sequencing analysis and radiomics. The GO and KEGG enrichment analysis was applied to explore the potential pathways.

## 2. Methods

### 2.1. Imaging Data of Patients with Bladder Cancer

The TCIA website (The Cancer Imaging Archive) hosts large volumes of cancer medical images that are deidentified and made publicly available for download. The data are organized into “collections,” such as patient imaging data associated with one disease (such as lung cancer), type of image (such as MRI and CT), or research topic (such as digital histopathology). DICOM is the main file format utilized by TCIA for radiology images. Furthermore, there are supplementary data available, including patient results, specifics of treatment, genomics, and expert evaluations. This study acquired 82 CT scans of patients with bladder cancer from the TCIA dataset, which is associated with the TCGA database.

### 2.2. The RNA-Sequencing from the TCGA Database

The study collected RNA-sequencing data and relevant clinical data on bladder cancer from The Cancer Genome Atlas (TCGA) database.

### 2.3. Image Segmentation

The segmentation of CT images, a critical step in our study, was performed by using a semiautomated method to delineate the regions of interest (ROIs) corresponding to the bladder cancer tumors. Each ROI was carefully reviewed and adjusted by two experienced radiologists to ensure accuracy, with discrepancies resolved by consensus.

### 2.4. Feature Extraction

Following segmentation, radiomics features were extracted from the delineated ROIs using “PyRadiomics” for Python. This comprehensive feature extraction process involved calculating a variety of features, including shape, intensity, texture, and wavelet-based features, to capture the tumor's phenotypic characteristics. The feature extraction parameters were set as follows: list key parameters, e.g., “bin width = 25 and resampling voxel size = 1 × 1 × 1 mm^3^,” based on best practices in the literature to ensure robustness and reproducibility of the feature set.

### 2.5. Feature Preprocessing and Selection

A feature preprocessing process consists of two steps: step 1 is to remove outliers and nulls and Step 2 is to normalize values in order to remove the dimensionality effect. The selection of features is one of the most crucial steps for better generalizing models, since high-dimensional data are often cluttered with irrelevant features, which can cause overfitting. Consequently, the variable space becomes simpler, and the variables are independent of one another. The final step is to construct radioactive features based on selected features by using AdaBoost cross-validation with leave-one-out.

### 2.6. Differentially Expressed Analysis in Bladder Cancer Cohort

The Limma package of the R language was used to analyze differential expression. An adjustment was made to the *P* values in TCGA in order to correct for false positives. To identify variations in mRNA expression, a threshold of “adjustable *P* < 0.01 and log_2_ (fold change) >2 or log_2_ (fold change) <−2” was utilized. While a log_2_ fold change (FC) of 1 (equivalent to a twofold change) is commonly used to denote statistical significance, we opted for a more stringent threshold to ensure the biological relevance of our findings. A log_2_ FC greater than 2 or less than −2 indicates a fourfold change in expression, highlighting genes with potentially greater biological impact and reducing the likelihood of identifying changes due to random variation or minor fluctuations in gene expression.

### 2.7. The Pathway Enrichment Analysis

The data underwent a functional enrichment analysis to validate the potential roles of the targets. GO (Gene Ontology) is a widely used tool to annotate genes with functions, such as molecular functions, biological pathways, and cellular components. Examining KEGG enrichment is a useful method for understanding gene function and genome function at a broad level. Enrichment analysis using GO and KEGG was conducted in the R programming environment.

### 2.8. Construction of Radiomics Signature through Feature Selection

The lasso technique was employed for regression analysis on data with many variables to identify the most valuable predictive characteristics from the initial dataset. The selection of the regularization parameter, *λ*, in lasso regression is critical as it determines the extent of the penalty applied to the features. To select an optimal *λ*, we utilized a cross-validation approach, specifically the 10-fold cross-validation method. This method involves dividing the dataset into ten parts, training the model in nine parts, and validating it in the remaining part. This process is repeated ten times, with each part serving as the validation set once. The optimal parameter was selected based on the *λ* value that produced the lowest cross-validation error. This approach ensures that the chosen *λ* is not only effective in minimizing the prediction error but also prevents overfitting by not overly penalizing the model, thereby preserving the predictive power of important features.

### 2.9. Creating a Personalized Forecasting Algorithm

To create a personalized prediction model using clinical information and RNA expression data, we conducted Cox regression analysis. First, a single-variable Cox regression analysis was conducted to identify characteristics with possible prognostic significance. Variables with a significance level below 0.05 in this initial examination were taken into account for incorporation in the multivariable model. Subsequently, we employed a stepwise selection process, considering both forward selection and backward elimination, to refine the list of variables included in the final model. This approach ensured that the final model contained only variables that significantly contributed to the prediction of patient outcomes, thereby enhancing the model's specificity and generalizability.

### 2.10. Statistical Analysis

Statistical analyses were performed in R, making use of its “radiomics” package for feature extraction and “survival” for survival analysis. Python was used for image processing tasks, by employing the PyRadiomics library for extracting radiomic features and scikit-image for image segmentation and preprocessing. The survival package in R allows for the execution of Kaplan–Meier survival analysis and log-rank tests. Log-rank tests were carried out to assess the statistical discrepancies in survival probabilities depicted in the Kaplan–Meier curves. This technique involves comparing the actual survival results with the predicted results assuming there is no distinction between the groups. A *p* value below 0.05 was deemed to be statistically significant. The relationship between the risk of survival and HR was assessed through the Spearman correlation test and the Cox proportional hazards model. We determined statistical significance by performing a rank sum test on the two datasets. A *p* value below 0.05 was deemed to be statistically significant. To mitigate the risk of false positives, we applied the Bonferroni correction method. This method entails modifying the importance level by dividing the standard *p* value of 0.05 by the total number of tests conducted.

## 3. Results

### 3.1. The Basic Information of 82 Bladder Cancer Patients

**Figure 1:**
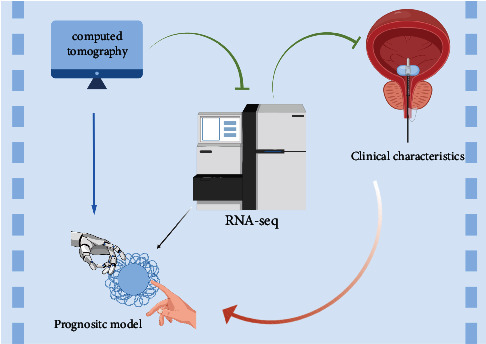
The mechanism diagram shows the process of analysis.

In this work, a total of 82 bladder cancer patients were involved in the analysis from the TCGA dataset. Out of the group, there were 28 individuals with bladder cancer who were younger than 65 and 54 individuals with bladder cancer who were older than 65. In addition, a total of 20 bladder cancer patients were female and a total of 62 bladder cancer patients were male. All the bladder cancer patients were involved in high grade. In terms of stage, there were 27 patients with bladder cancer in stage II, 31 patients with bladder cancer in stage III, and 24 patients with bladder cancer in stage IV. For the T stage, 1 bladder cancer patient was involved in the T0 stage, a total of 6 bladder cancer patients were in the T2 stage, a total of 8 bladder cancer patients were in the T2a stage, a total of 14 bladder cancer patients were in the T2b stage, a total of 10 bladder cancer patients were in the T3 stage, a total of 16 bladder cancer patients were in the T2a stage, a total of 13 bladder cancer patients were in the T3b stage, 1 bladder cancer patient was in the T4 stage, a total of 8 bladder cancer patients were in the T4a stage, and a total of 5 bladder cancer patients were in the unknown T stage. In terms of the N stage, a total of 47 bladder cancer patients were in the N0 stage, a total of 9 bladder cancer patients were in the N1 stage, a total of 14 bladder cancer patients were in the N2 stage, a total of 11 bladder cancer patients were in the NX stage, and 1 bladder cancer patient was in the unknown N stage. For the M stage, a total of 42 bladder cancer patients were in the M0 stage, a total of 3 bladder cancer patients were in the M1 stage, and a total of 37 bladder cancer patients were in the MX stage.  Figure 1 introduces the process of this work in detail.

### 3.2. Results from Imaging Using Computed Tomography in Patients with Bladder Cancer

**Figure 2:**
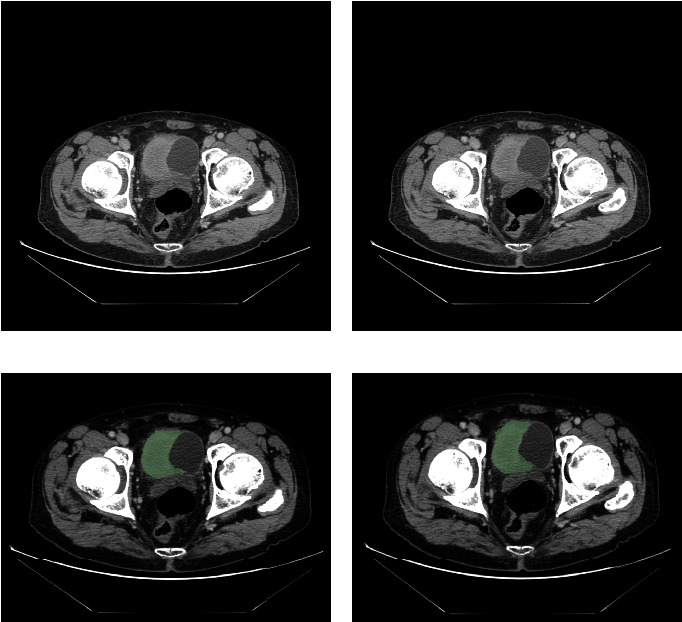
(a, b) The CT image of a 65-year-old bladder cancer patient and (c, d) the ROI of the CT image of a 65-year-old bladder cancer patient.

**Figure 3:**
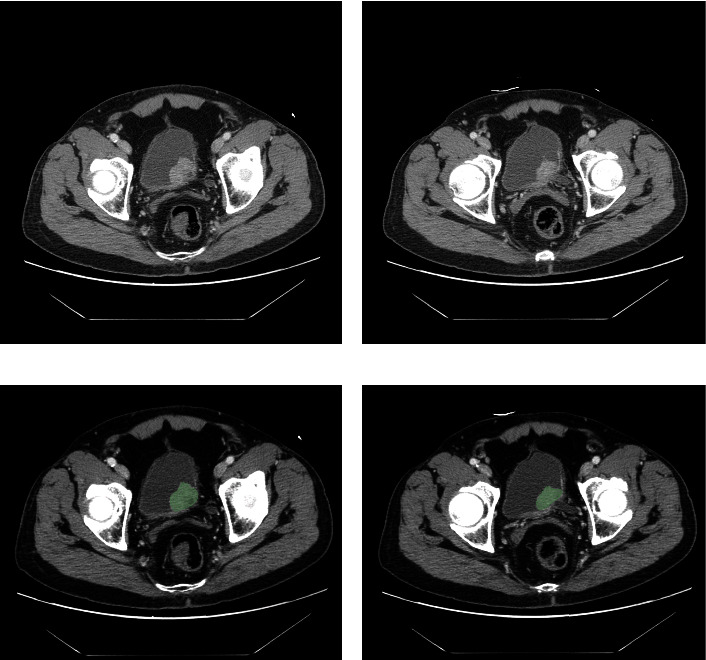
(a-b) The image shows the CT of a 64 years old bladder cancer patients; (c-d) the Identification of ROI in CT image.

In the first step, all CT imaging results of 82 bladder cancer patients were uploaded to the 3D Slicer software, which allows to visualize, process, segment, register, and analyze medical, biomedical, and other 3D images and meshes for free and open source. Here, the CT of two patients with bladder cancer is shown in the figure.  Figure 2 shows a male bladder cancer patient with high grade, 65 years old, stage III, T3a stage, N0 stage, and M0 stage (Figures 2(a) and 2(b)). In addition, Figure 3 shows a male bladder cancer patient with high grade, 64 years old, T2a stage, M0 stage, and N0 stage (Figures 3(a) and 3(b)). In the next step, we outline the ROI (region of interest), as well as each slice of the CT, so that we can extract the three-dimensional features of each bladder cancer image. The main component of the tumor is believed to be its ROI. The tumor tissue was reconstructed using a 2 mm dilation algorithm in this study. An NRRD format file will be produced for each sample after it is sketched and exported (Figures 2(c), 2(d), 3(c), and 3(d)).

### 3.3. Extraction of Imaging Features in Bladder Cancer Patients and Radiomics Signature Construction

**Figure 4:**
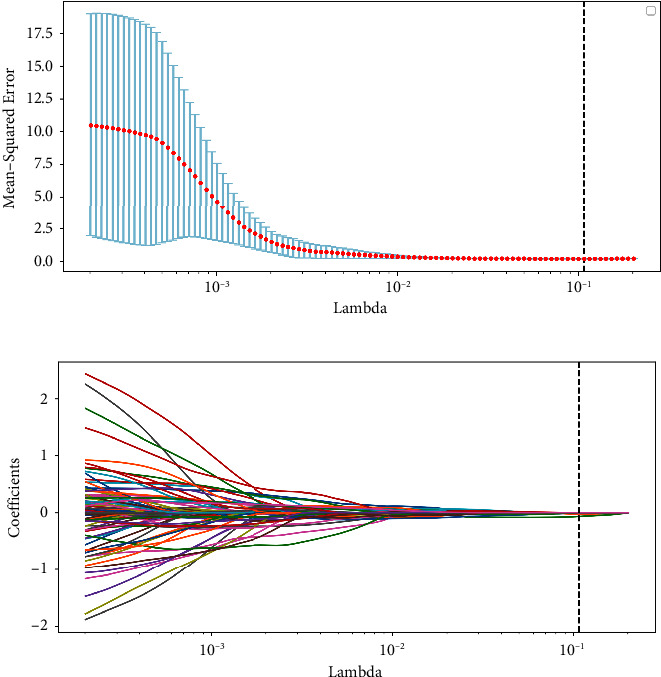
(a, b) The lasso regression analysis was applied to select the imaging features of CT.

**Figure 5:**
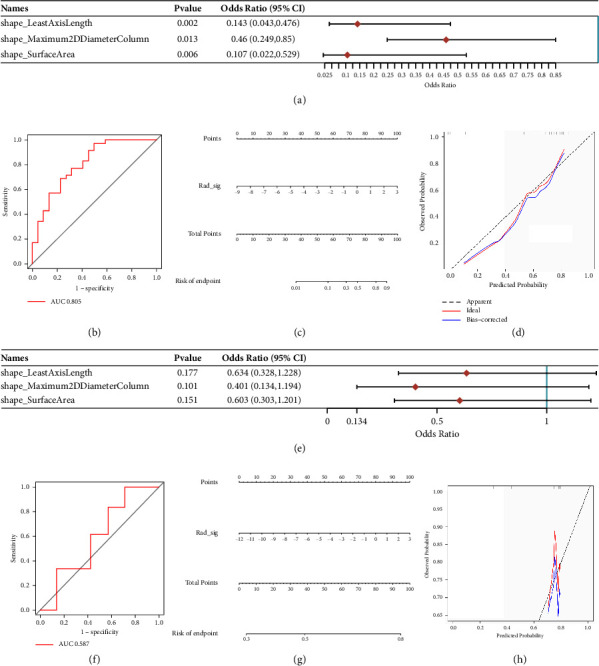
(a) The forest plot demonstrated the imaging features in the train set, (b) the ROC curve demonstrated the predictive value of the radiomics signature in the train set, (c) the nomogram of radiomics signature in the train set, (d) the calibration curve reveals the predictive value of the nomogram in the train set, (e) the forest plot demonstrated the imaging features in the test set, (f) the ROC curve demonstrated the predictive value of radiomics signature in the test set, (g) the nomogram of radiomics signature in the test set, and (h) the calibration curve reveals the predictive value of the nomogram in the test set.

After analyzing the data, we identified a combined total of 129 visual characteristics. Initially, the group of patients with bladder cancer was split into a training set and a testing set, with a ratio of 7:3. Then, we performed the lasso regression analysis. Lasso logistic regression reduced 129 features to four potentially predictive factors and one predictor with a nonzero coefficient (Figures 4(a) and 4(b)). Model = 0.11 + (−1.019) *∗* Least Axis Length + (0.403) *∗* Maximum 2D Diameter Column + (−1.019) *∗* Surface Area. The forest plot in the train cohort and test cohort demonstrated that these 3 features may be important factors in the bladder cancer cohort (Figures 5(a) and 5(e)). The AUC score of the ROC curve in the train set was 0.805. The AUC score of the ROC curve in the test set was 0.587 (Figures 5(b) and 5(f)). Furthermore, we created the nomogram for both the training and testing datasets. In the training and testing datasets, the nomogram's C-index was 0.805 (95% CI: 0.774–0.836) and 0.587 (95 CI: 0.475–0.699), respectively, as shown in Figures 5(c) and 5(g). The nomogram showed strong predictive ability in both the training and testing sets, as indicated by the calibration curve (Figures 5(d) and 5(h)).

### 3.4. The Genes That Are Expressed at Varying Levels in the Bladder Cancer Cohort

**Figure 6:**
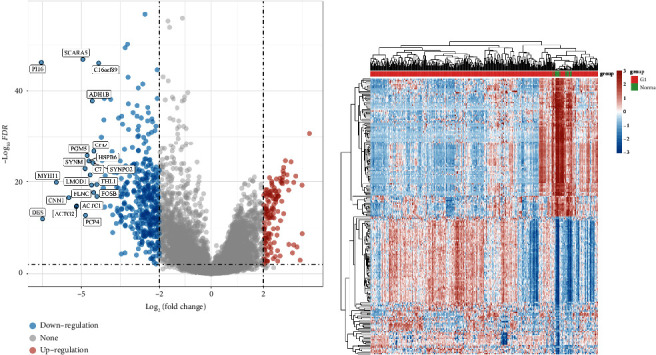
(a) The volcano map demonstrated the differentially expressed genes in the bladder cancer cohort and (b) the heatmap demonstrated the differentially expressed genes in the bladder cancer cohort.

To investigate the genes closely linked to bladder cancer, we conducted differential expression analysis comparing the normal group with the bladder cancer cohort. The findings indicated that 546 genes were identified as differentially expressed, with 136 genes showing an increased expression and 410 genes showing a decreased expression (Figure 6(a)). The heatmap illustrated the genes that were expressed differently in the bladder cancer group compared to the normal group (Figure 6(b)).

### 3.5. The Potential Routes Linked to Genes with Varying Expression Levels in the Bladder Cancer Group

**Figure 7:**
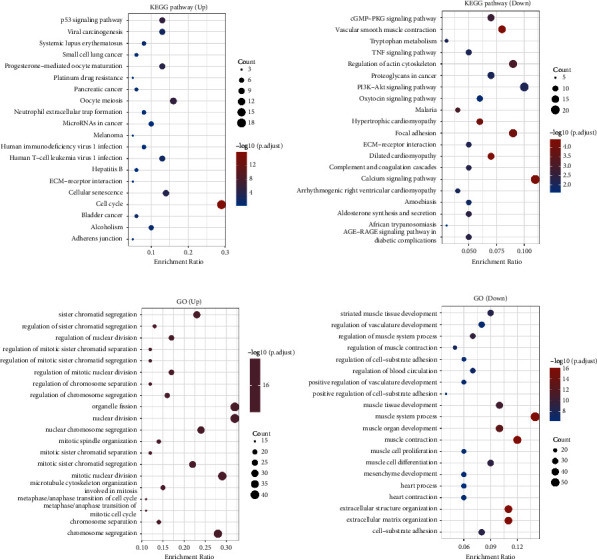
(a) The upregulated KEGG enrichment pathways, (b) the downregulated KEGG enrichment pathways, (c) the upregulated GO enrichment pathways, and (d) the downregulated GO enrichment pathways.

We then assessed the possible routes linked to the genes that are expressed differently. In KEGG enrichment analysis, the pathways with the highest enrichment of upregulated genes are the p53 signaling pathway, viral carcinogenesis, platinum drug resistance, and oocyte meiosis as shown in Figure 7(a). The pathways that are most enriched in downregulation include cGMP-PKG signaling, vascular smooth muscle contraction, TNF signaling, and tryptophan metabolism (Figure 7(b)). The pathways most upregulated for GO enrichment analysis include sister chromatid segregation, regulation of sister chromatid segregation, regulation of nuclear division, and regulation of mitotic sister chromatid segregation (Figure 7(c)). Furthermore, the pathways with the most downregulated activity include the development of striped muscle tissue, the regulation of blood vessel development, the regulation of muscle system processes, and the regulation of muscle contractions as shown in Figure 7(d).

### 3.6. The Integrated Predictive Model Based on the Radiomics Signature and Gene Signature

**Figure 8:**
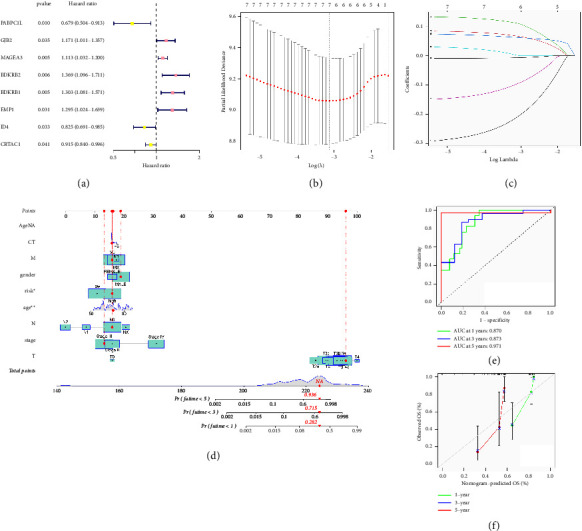
(a) In the univariate Cox regression analysis, the results demonstrated that 8 differentially expressed genes were associated with the prognosis of bladder cancer patients, (b, c) the lasso regression analysis in bladder cancer cohort, (d) the nomogram was constructed based on radiomics signature and gene signature, (e) the ROC curve shows the predictive value of radiomics signature and gene signature, and (f) the calibration curve shows the predictive value of the nomogram.

After analyzing the previous data, we were able to identify the genes that were expressed differently in the bladder cancer group. Following this, an investigation will be conducted to identify the genes that have a strong correlation with the survival outlook of individuals with bladder cancer. Initially, we conducted a univariate Cox regression analysis which revealed that 8 genes with differential expression were linked to the prognosis of individuals with bladder cancer (Figure 8(a)). Then, the lasso regression analysis was performed to further explore the prognosis-related genes. The multivariate Cox regression analysis revealed that four genes could potentially play a crucial role in determining the prognosis of patients with bladder cancer (Figures 8(b) and 8(c)). We also constructed the gene signature by using the following formula: PABPC1L *∗* −0.30049004562043 + MAGEA3 *∗* 0.0885112110183904 + BDKRB2 *∗* 0.244350992743845 + ID4 *∗* −0.168007057831507. To improve the outlook for individuals with bladder cancer, we developed a nomogram using clinical data, radiomics signature, and gene signature (Figure 8(d)). The nomogram we developed was created by analyzing a range of clinical and radiomic factors, such as age, sex, tumor grade, stage, and specific radiomic characteristics derived from CT images. Each variable was assigned a weight in the nomogram based on its prognostic significance, determined through Cox regression analysis. Variables such as tumor grade and stage, which are pivotal in bladder cancer prognosis, were given significant weights, reflecting their importance in the model. The ROC graph illustrated AUC scores of 0.870, 0.873, and 0.971 for 1-year, 3-year, and 5-year, respectively, as shown in Figure 8(e). In addition, the calibration curve indicated that the nomogram has strong predictive accuracy in the bladder cancer group (Figure 8(f)).

## 4. Discussion

According to GLOBOCAN statistics, bladder cancer makes up 3% of global cancer cases and is especially common in developed nations [15]. According to data from the United States, bladder cancer ranks as the sixth most prevalent type of cancer [16]. Individuals aged 55 and above account for 90% of bladder cancer cases, with men being four times more susceptible to the disease compared to women [17]. In the United States, the 5-year survival rate for patients with metastatic cancer is only 5%, which is significantly lower than the overall 5-year survival rate of 77%. Therefore, it is very urgent to explore the promising biomarkers for the better prediction of bladder cancer and for seeking diagnosis and treatment for the bladder cancer patients [18].With the development of multiple images, cancer can be diagnosed easily [19]. A CT scan is a frequently used technique for diagnosing bladder cancer [20]. Early detection of bladder cancer before surgery can lead to accurate preoperative staging and early detection of recurrence postoperatively. Conventional CT scans necessitate additional storage and processing time as they encompass extensive details regarding the human anatomy [21]. Hence, proper segmentation of CT images is crucial for improving the diagnosis and treatment of individuals with bladder cancer [22]. In this work, we first obtained the CT images of 82 bladder cancer patients. Then, we outlined the ROI and each CT slice in order to extract the three-dimensional features from each image of bladder cancer. Finally, we successfully constructed the radiomics signature. The ROC curve and nomogram demonstrated that the radiomics signature shows good predictive value in bladder cancer patients. Recently, as bioinformatics analysis has advanced quickly, an increasing number of research projects are investigating biomarkers for cancer patients. A radiological model was created to forecast the survival of cervical cancer patients using medical images, statistical models, and machine learning, as demonstrated in a previous study. In addition, another study demonstrated that deep learning algorithms based on CT images and ceramide glycosylation have important application value in bladder cancer diagnosis [23]. In the selection of AdaBoost (adaptive boosting) for the cross-validation in feature selection, our decision was motivated by several factors that align with the goals of our study. AdaBoost is renowned for its capacity to convert a series of weak classifiers into a strong classifier, making it particularly suitable for our dataset where the predictive power of individual features might be modest [24]. Through iterative processes, this algorithm rectifies errors made by weak classifiers and modifies the weights of misclassified instances, thereby enhancing the model's capacity to generalize beyond the training data to new data [25].

Furthermore, due to advancements in next-generation sequencing technology, RNA-Seq technology can now be used in a wider range of applications. RNA sequencing can provide cancer patients with a variety of biomarkers to aid in their diagnosis and treatment. Following this, we conducted an analysis of genes with varying expression levels and developed a prognostic prediction model based on 4 genes using Cox and lasso regression techniques. Obtaining biomarkers with various indicators, including clinical characteristics, radiomics signature, and gene signature, is crucial due to their strong predictive value in cancer patients. In this study, we have effectively developed a prognostic prediction model by integrating the radiomics signature with the gene signature. The nomogram, along with the ROC curve, further confirmed the model's precision. The ability to accurately stage bladder cancer with CT technology will gradually improve as the technology continues to be updated and improved. As deep learning algorithms become more sophisticated, CT scanning may become a routine screening for bladder cancer in the future. It is believed that radiomic features will also become an effective target for bladder cancer treatment in the future, since these features are closely related to prostate cancer development and prognosis. For KEGG enrichment analysis, the increase in activity of the p53 signaling pathway, viral-induced cancer development, resistance to platinum drugs, and pathways related to oocyte maturation indicate an intricate interaction involving genetic changes, environmental influences, and resistance to chemotherapy in bladder cancer. These pathways are pivotal in cell cycle regulation, DNA damage response, and apoptosis, indicating their critical roles in tumor development and response to treatment. On the other hand, the suppression of pathways such as cGMP-PKG signaling, vascular smooth muscle contraction, TNF signaling, and tryptophan metabolism could indicate the tumor microenvironment's adjustment, facilitating tumor development and avoiding immune detection.

The integration of clinical, radiomics, and gene data represents a significant advancement in developing a multifaceted prognostic model for bladder cancer. However, the interplay between these data types is complex and warrants further discussion. Clinical data provide a foundational understanding of patient health and disease characteristics, while radiomics and genetic data offer deeper insights into the tumor's phenotypic and molecular landscape. While our study highlights the potential of CT technology and deep learning algorithms in improving the accuracy of bladder cancer staging, we recognize the challenges in generalizing these findings universally. Variations in healthcare infrastructure, access to advanced diagnostic tools, and population genetics can influence the applicability and effectiveness of these technologies. Furthermore, the diversity in patient demographics underscores the need for models that are robust across different ethnicities, ages, and genders. In order to increase the generalizability of our findings, upcoming research should strive to incorporate a wider range of patients and take into account the differences in healthcare delivery systems. This method not only improves the applicability of the results but also offers a more thorough understanding of the possible obstacles and aids in incorporating these technologies in different situations. It is crucial for advancing personalized medicine and ensuring that innovations in cancer diagnosis and treatment are accessible and effective for all segments of the population, regardless of geographical or socioeconomic status. Moreover, the integration of these technologies into clinical practice involves overcoming regulatory, ethical, and logistical hurdles. It is imperative to conduct further research to validate these approaches in diverse populations and settings and to continuously monitor their performance in real-world clinical scenarios.

In conclusion, it has been demonstrated that deep learning can be applied to CT images of bladder cancer to effectively segment lesions. CT images based on algorithmic algorithms are significantly more accurate than ordinary imaging examinations for staging bladder cancer. In addition, it was discovered that bladder cancer tissue harbors genes associated with prognosis, which can effectively forecast patient outcomes. The prognostic prediction model, based on radiomics signature and gene signature, effectively forecasts the outcome for individuals with bladder cancer.

## Data Availability

The data used to support the findings of the study are available from the corresponding author upon request.
